# Comparison between the timing of the occurrence of taste sensitivity changes and short-term memory decline due to aging in SAMP1 mice

**DOI:** 10.1371/journal.pone.0248673

**Published:** 2021-03-23

**Authors:** Masataka Narukawa, Suzuka Takahashi, Aya Kamiyoshihara, Kentaro Matsumiya, Takumi Misaka

**Affiliations:** 1 Graduate School of Agricultural and Life Sciences, The University of Tokyo, Tokyo, Japan; 2 Department of Food and Nutrition, Kyoto Women’s University, Kyoto, Japan; 3 Graduate School of Agriculture, Kyoto University, Kyoto, Japan; iBiMED - Institute of Biomedicine, PORTUGAL

## Abstract

Several studies have suggested that cognitive impairment affects taste sensitivity. However, the mechanism behind this is still unclear. In this study, we focused on short-term memory. Using senescence-accelerated mouse prone 1 (SAMP1) mice, we compared whether the effects of aging are observed earlier in taste sensitivity or short-term memory. We used 8-week-old mice as the young group, and 70- and 80-week-old mice as aged groups. Taste sensitivity was evaluated using a 48-hour two-bottle preference test, and short-term memory was evaluated using the Y-maze test. SAMP1 mice showed apparently changes in taste sensitivity at 70-weeks-old. However, the influence of aging on spontaneous alternation behavior, which is indicative of short-term memory alterations, was not observed in 70-week-old mice. At 80-weeks-old, the influence of aging was observed, and spontaneous alternation behavior was significantly decreased. This suggests that age-dependent changes in taste sensitivity occur prior to short-term memory function decline. In addition, there was no significant influence of aging on the mRNA expression of long-term potentiation-related genes in the hippocampus of 80-week-old mice. Therefore, the age-related decline of short-term memory may not affect taste sensitivity.

## Introduction

Health in the elderly is becoming an increasingly important social issue as a result of the progressively aging populations in most developed countries. A balanced nutritional diet is necessary to maintain good health. The sense of taste is a chemical sensation that primarily detects the nutrients present in food. Maintaining the sense of taste is important to ensure that older people have a balanced nutritional diet [1].

Taste sensitivity has been reported to gradually change in an age-dependent manner in humans in numerous studies [2–4]. However, the molecular mechanisms underlying this phenomenon remain unclear. We have reported that the changes in taste sensitivity due to aging are caused by factors other than those responsible for deterioration of taste detection systems in the oral cavity [5, 6]. On the other hand, age-related changes in taste function have been associated with changes in neuronal circuits [7]. This suggests that functional changes in the central nervous system may affect to taste sensitivity. Furthermore, studies have reported a decline in taste sensitivity in patients with Alzheimer’s disease [8–11]. Alzheimer’s disease is a syndrome caused by a number of progressive illnesses that affect cognitive capacity in adults aged 65 years and older [8]. These results suggest that cognitive function affects taste sensitivity. However, the effects of such changes in brain function on taste sensitivity remain unknown.

Cognitive functions are composed of various abilities, including perception, learning, and memory [12]. Among cognitive functions, short-term memory ability can be evaluated non-invasively and simply using the Y-maze test. To investigate whether cognitive impairment affects taste sensitivity, we focused on short-term memory as representative for cognitive functions and compared whether the effects of aging are observed earlier in taste sensitivity or short-term memory. We here employed senescence-accelerated mouse (SAM) which is an inbred strain that shows hastened aging compared to a general mouse [13]. We have previously reported that taste sensitivities to salty and bitter substances were increased in older mice (70-weeks-old) compared to young mice (8-weeks-old) in the SAMP1 strain [6]. Thus, short-term memory and taste sensitivity to salty and bitter substances in 70- and 80-week-old mice were measured by behavioral experiments and were compared with those of young SAM mice (8-weeks-old). After behavioral experiments, we observed the expression levels of several mRNAs, including those of long-term potentiation-related genes, in the hippocampus.

## Materials and methods

### Materials

NaCl was purchased from Kanto Chemical (Tokyo, Japan). Denatonium benzoate (denatonium) was purchased from Sigma. All other reagents were of analytical grade and obtained from standard suppliers.

### Animals and experimental procedures

All experiments were performed in accordance with protocols approved by the University of Tokyo Animal Care Committee (Approval Number: P18-075). SAM consists of nine senescence-prone inbred strains (SAMP). Among the commercially available SAMP1, P6, P8, and P10 strains, the SAMP1 strain was used because it had the highest grading score of senescence (8.72) at eight months of age [14]. Seven-week-old male mice were purchased from Japan SLC (Hamamatsu, Japan) and were housed at the University of Tokyo Animal Care Facility until the beginning of the behavioral experiments. The mice had *ad libitum* access to standard laboratory chow and distilled water. The surrounding temperature and humidity were maintained at 23°C and 55%, respectively, with a 12-h/12-h light/dark cycle (lights switched on at 0800 h).

We started the behavioral experiments using mice that were 70-weeks-old. These mice were subjected to the same behavioral experiments at 80-week-old to assess the progression of aging. We employed 8-week-old mice as the young control group, and the behavioral experiments were simultaneously performed using 8-week-old mice. In total, 25 and 21 mice were allocated to the 8-week-old and 70-week-old groups, respectively. At 80 weeks of age, 14 of the 21 mice in the 70-week-old group had survived. SAMP1 mice with normal shapes and feeding behaviors were used.

The duration of the behavioral experiments was approximately two weeks. Initially, a grip strength test was performed to assess the decline in muscle strength due to aging in the SAMP1 mice. Next, the Y-maze and 48-h two-bottle preference tests were used. After the behavioral experiments, the mice were dissected under anesthesia to extirpate the hippocampus.

### Grip strength test

Before the beginning of the behavioral tests, the grip strength of the forelimb was measured using a Grip Strength Meter (GPM-101B; Melquest, Toyama, Japan). As the mouse grasped the bar, the peak pull force in grams was recorded on a digital force transducer. The measurements were repeated five times. Excluding the maximum and minimum values, the average of the three values was calculated as the grip strength.

### Y-maze test

The Y-maze had three arms (400 mm deep, 200 mm high, 30 mm wide at the bottom, and 100 mm wide at the top) at angles of 120° (Shinfactory, Fukuoka, Japan). Mice were placed at the end of one arm and allowed to move freely for 10 min. The frequency of arm entry was counted manually to calculate the total number of entries. An alternation was defined as consecutive entries into all three arms (e.g., 1, 2, 3, or 1, 3, 2). The alternation ratio was calculated as the number of alternations (entries into three different arms consecutively) divided by the total number of possible alternations (i.e., the number of arms entered minus 2) and multiplied by 100.

### 48-hour two-bottle preference test

Mice were caged individually and given 48 h of access to two bottles, one containing deionized water and the other containing tastant solution. After 24 h, the bottle positions were switched to avoid positional effects. The preference ratio was calculated as follows: tastant intake/total fluid intake (tastant intake + water intake). The tastant solutions for the two-bottle preference test were 300 mM NaCl (salty taste) and 1 mM denatonium (bitter taste). The tastant solutions were presented in an ascending series of concentrations. The mice were habituated by presenting two bottles that contained water for only one week before starting the preference test, and it was confirmed that the mice drank water evenly from both the bottles. Apart from the mice that died during the preference test, each mouse was tested for all solutions.

### Real-time polymerase chain reaction

After the behavioral experiments, the mice were dissected under anesthesia (a mixture of medetomidine [0.3 mg/kg], midazolam [4 mg/kg], and butorphanol [5 mg/kg]) to remove the hippocampus. Total RNA from the hippocampus was extracted using RNeasy Mini Columns (Qiagen, Venlo, The Netherlands). Total RNA was extracted from six randomly selected samples from the collected samples. Genomic DNA digestion was performed using the RNase-free DNase Set (Qiagen). First-strand cDNA was generated from total RNA by reverse transcription (Superscript IV reverse transcription kit; Thermo Fisher Scientific, Waltham, MA, USA). The mRNA transcript levels were determined by quantitative polymerase chain reaction (qPCR; ABI Prism 7000 Sequence Detection System; Thermo Fisher Scientific). PCR amplification was performed using the SYBR green PCR system (Thermo Fisher Scientific). The PCR primers used in the SYBR green PCR system are described in S1 Table. The qPCR reactions were performed using the following protocols: for the SYBR green PCR system, 50°C for 2 min, 95°C for 2 min, 45 cycles of 95°C for 15 s and 60°C for 1 min, and dissociation step (95°C for 15 s, 60°C for 20 s, and 95°C for 15 s). The delta-delta method was used for relative quantification [15]. The mRNA expression of *Gapdh* was used as an internal control.

### Statistical analysis

The results were expressed as the mean ± standard error of the mean (SEM). All statistical analyses were performed using GraphPad Prism 6 software (GraphPad Software, CA, USA). Differences were evaluated using a one-way analysis of variance (ANOVA) coupled with Tukey’s posthoc test or *t*-test. For all analyses, differences with *p*-values lower than 0.05 were considered significant.

## Results

### Changes in aging phenotypes over time

**Fig 1:**
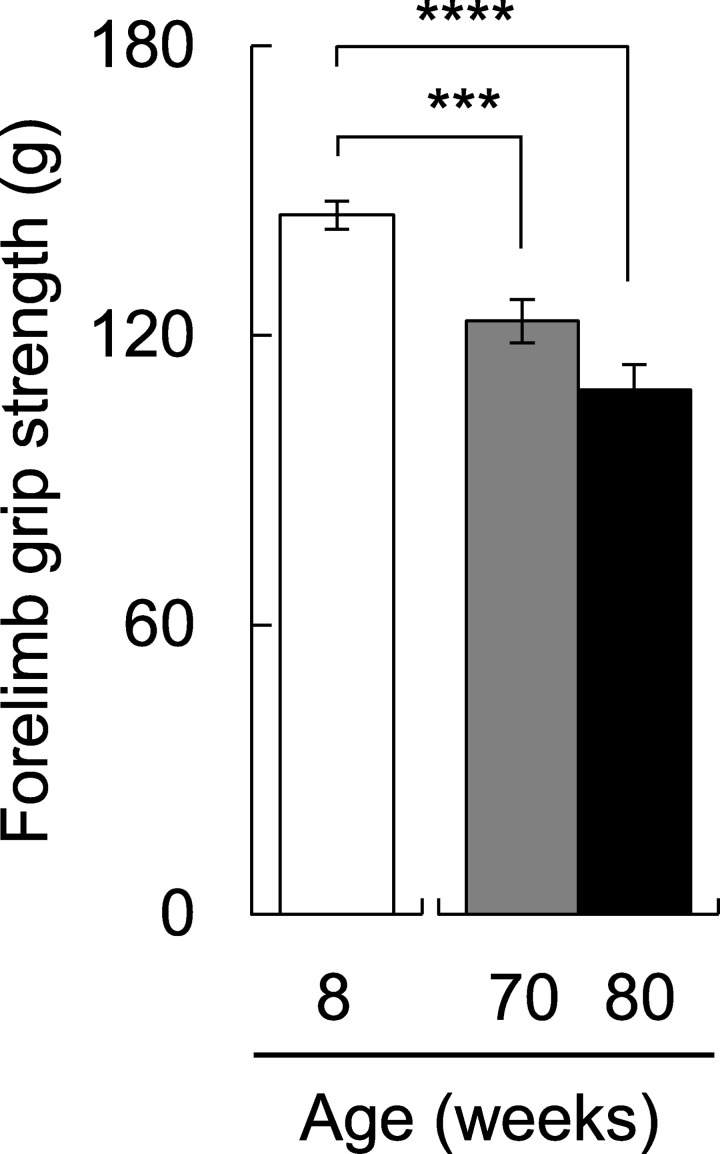
Change of muscle strength due to aging. Forelimb grip strength of 8-week-old (n = 25), 70-week-old (n = 21), and 80-week-old mice (n = 14). Grip strength significantly decreased with age *(F*_*(2*,*57)*_ = 20.4, *p* < 0.0001; one-way ANOVA). *** *p* < 0.001 and **** *p* < 0.0001 (Tukey posthoc test).

**Fig 2:**
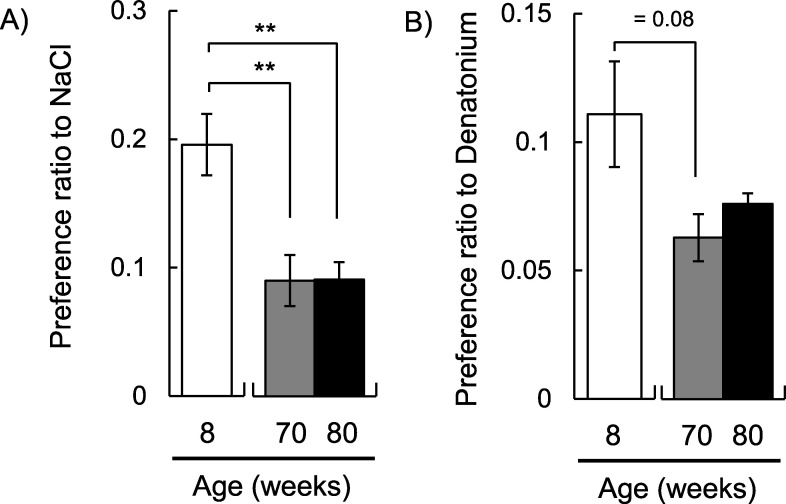
Taste sensitivity changes due to aging. The preference ratios for (A) salty (300 mM of NaCl) and (B) bitter taste (1 mM of denatonium) to water are shown (8-week-old mice for n = 24–25, 70-week-old mice for n = 19, 80-week-old mice for n = 13). The preference ratio to NaCl significantly decreased with age (*F*_*(2*,*54)*_ = 8.34, *p* < 0.001; one-way ANOVA). The preference ratio to denatonium tended to decrease with age *(F*_*(2*,*53)*_ = 2.65, *p* = 0.08; one-way ANOVA). ** *p* < 0.01 (Tukey posthoc test).

**Fig 3:**
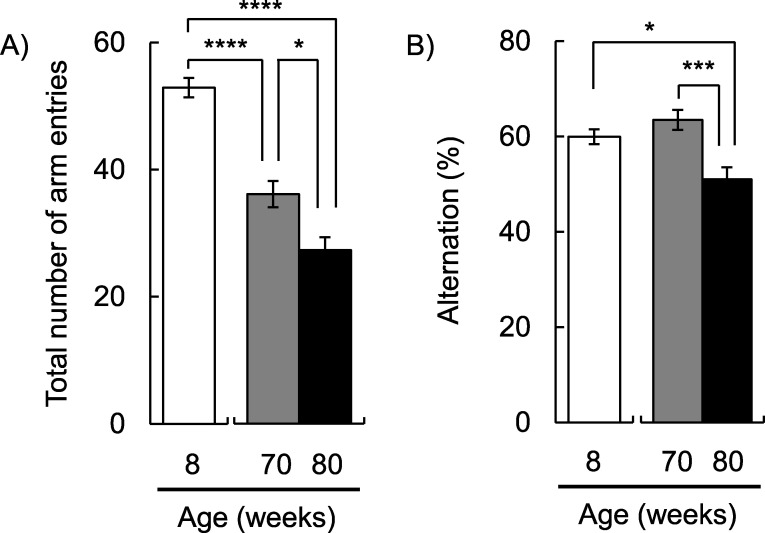
Short-term memory changes due to aging. (A) The total number of arm entries and (B) the alteration ratio are shown (8-week-old mice for n = 25, 70-week-old mice for n = 21, 80-week-old mice for n = 14). The total number of arm entries and alternation ratio significantly decreased with age (*F*_*(2*,*57)*_ = 44.0, *p* < 0.0001 for the total number of arm entries, *F*_*(2*, *57)*_ = 7.77, *p* < 0.001 for the alternation ratio; one-way ANOVA). * *p* < 0.05, *** *p* < 0.001 and **** *p* < 0.0001 (Tukey posthoc test).

In order to check the decline in physical function due to aging in SAMP1 mice, we measured muscle strength. In the 70-week-old mice, muscle strength was significantly declined compared to the 8-week-old mice (*p* < 0.001; Fig 1). As we were able to observe the characteristic phenotype of aging in SAMP1 mice, the change in taste sensitivity to salty and bitter was measured. Regarding salty taste, the preference ratio to NaCl in 70-week-old mice was significantly decreased compared to the 8-week-old mice (*p* < 0.01; Fig 2A). Regarding bitter taste, we confirmed that the preference ratio to denatonium apparently decreased in 70-week-old mice compared to 8-week-old mice (*p* = 0.08; Fig 2B). Next, we compared short-term memory ability using the Y-maze test. In 70-week-old mice, there was a significant decrease in the total number of arm entries compared to the 8-week-old mice (*p* < 0.0001; Fig 3A). However, the alteration ratio in 70-week-old mice did not decrease compared to the 8-week-old mice (Fig 3B).

We conducted the same experiments using 80-week-old mice. Grip strength and preference ratio to NaCl in 80-week-old mice were lower than those of the 8-week-old mice (*p* < 0.001 for grip strength; *p* < 0.01 for preference ratio to NaCl; Figs 1 and 2A). The preference ratios to denatonium in 80-week-old mice were not significantly different, but they were lower than those in the 8-week-old mice (*p* = 0.33; Fig 2B). In the Y-maze test, the total number of arm entries was further decreased in the 80-week-old mice (*p* < 0.0001; Fig 3A). We also confirmed that the alteration ratio in 80-week-old mice was significantly decreased compared to that in 8-week-old mice (*p* < 0.05; Fig 3B). The total number of arm entries and the alteration ratio in 80-week-old mice were significantly lower than in 70-week-old mice (*p* < 0.05 for the total number of arm entries; *p* < 0.001 for the alteration ratio). These results indicate that the influence of aging appears earlier in taste sensitivity than in the memory function.

### Evaluation of the gene expression levels in the hippocampus

**Fig 4:**
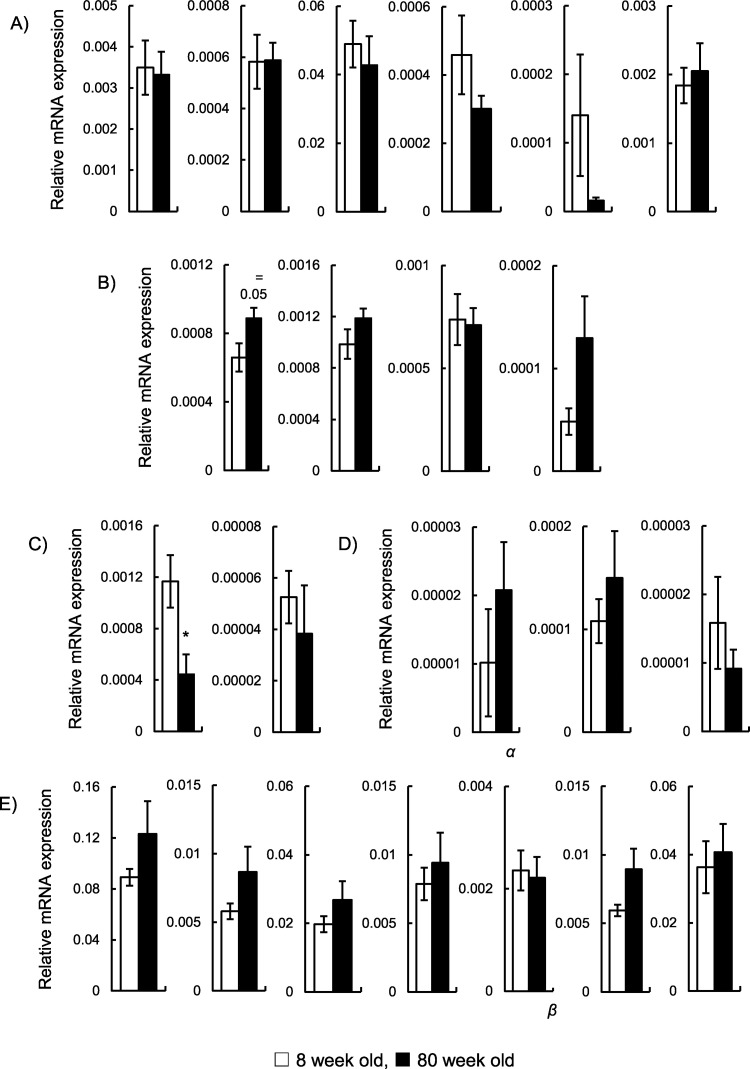
Comparison of mRNA expression in the hippocampus. Quantitative mRNA expression for (A) nerve growth factor, (B) NADPH oxidase, (C) nitric oxide synthase, (D) inflammatory, and (E) long-term memory store-related genes of 8-week-old (white column, n = 6) and 80-week-old mice (gray column, n = 6). * *p* > 0.05 (Welch’s *t*-test).

Because the hippocampus plays a key role in memory formation [16], we compared the mRNA expression of representative genes between the hippocampus of 8-week-old and 80-week-old mice in the hippocampus (Fig 4). We observed the mRNA expression levels of long-term potentiation-related genes and nerve growth factors that play a role in memory formation [17]. Because chronic inflammatory status is a feature of aging [18], mRNA expression of inflammation-related genes, including nicotinamide adenine dinucleotide phosphate (NADPH) oxidase and nitric oxide synthase, were also measured. A comparison of the gene expression levels revealed that the mRNA expression level of *eNos* (a member of nitric oxide synthase) significantly decreased with aging (*p* < 0.05; Fig 4C). Furthermore, mRNA expression levels of *p22*^*phox*^ (a subunit of NADPH oxidase) tended to increase with aging (*p* = 0.05, respectively; Fig 4B). However, there were no significant differences in the expression levels of the other genes, including long-term potentiation-related genes. This suggests that aging does not significantly affect the mRNA expression of long-term potentiation-related genes.

## Discussion

We have previously reported that changes in taste sensitivity due to aging are caused by factors other than those responsible for the deterioration of taste detection systems in the oral cavity [5, 6]. On the other hand, it has been reported that age-related changes in taste function are related to alterations in the neuronal circuits [7]. In addition, some studies reported that patients with Alzheimer’s disease show higher taste threshold than control subjects [8–11]. These suggest that functional changes in the central nervous system, such as cognitive function decline, may affect the age-dependent changes in taste sensitivity. In this study, we focused on short-term memory as a representative for cognitive functions and taste sensitivity changes and compared whether the effects of aging are observed earlier in taste sensitivity or short-term memory. We targeted short-term memory because short-term memory ability can be evaluated simply and non-invasively by using the Y-maze test.

In general, muscle strength and motor activity decline with age. Grip strength was decreased in the 70-week-old mice and was even lower in the 80-week-old mice. As the total number of arm entries provides a measure of general activity, it is considered that a reduced number of arm entries means reduced activity due to aging. In fact, the total number of arm entries decreased with age. The reduced physical functions mean that tested mice were aged sufficiently. However, spontaneous alternation behavior was not changed in 70-week-old mice. In contrast, 80-weeks-old mice showed signs of cognitive aging by significantly decreased spontaneous alternation behavior. It is considered that there was no apparent decrease in short-term memory ability at 70-week-old. On the other hand, we confirmed that the preference ratio to NaCl in 70-week-old mice was significantly decreased compared to the 8-week-old mice. Regarding denatonium preference, we confirmed not significantly but apparent decrease in 70-week-old mice compared to 8-week-old mice. Here, because a decrease in the preference ratio means that mice showed strong avoidance to these stimuli, it has the same meaning as an increase in taste sensitivity. Thus, we observed the changes in taste sensitivity at 70-week-old. This suggests that the decrease in taste preference apparently occurs earlier than that in the alternation ratio.

As the preference ratio to NaCl had declined in 70-week-old mice, little change was observed in 80-week-old mice. As the reason for this, it is considered that because the preference ratio to 300 mM NaCl at 70-week-old was sufficiently low, that at 80-week-old did not decrease further. Although a significant decrease in bitter preference has been reported [6], we were not able to confirm a significant decrease in the preference ratio to denatonium due to aging. One of the reasons is the concentration of denatonium. Because the taste intensity of denatonium was just strong, the significant difference may not have been observed. When the concentration is low (<1 mM), a clearer difference may be observed.

Generally, taste sensitivity is evaluated using a 48-h two-bottle preference test and/or a brief access test in rodents. When we evaluated the effect of aging on taste sensitivity in SAMP1 mice, we observed similar results to the salty and bitter taste in the two-bottle assay and brief access test [6]. Therefore, we employed the two-bottle test to evaluate taste sensitivity in the present study.

Cognitive function includes the domains of perception, memory, learning, attention, decision making, and language [12]. A Y-maze test is a simple experiment used in animal cognition experiments. Spontaneous alternation behavior of mice in a Y-maze is generally considered short-term memory [19]. Therefore, this test seems to reflect part of cognitive function. On the other hand, we have previously investigated the impact of cognitive impairment on taste sensitivity using an AD mouse model, *App*^*NL-G-F*^ mice [20]. There was no significant difference in taste sensitivity between wild type and *App*^*NL-G-F*^ mice. Furthermore, we have also reported that the intake of α-glycerophosphocholine, a precursor of the neurotransmitter acetylcholine, improved age-related decline in the gene expression levels of long-term potentiation in the hippocampus but not taste sensitivity [21]. These results suggest that age-related cognitive impairment may not greatly affect taste sensitivity. The mRNA expression of representative genes in the hippocampus plays a key role in memory formation [16]. No significant change was observed in the expression of long-term potentiation-related genes, supporting the notion that short-term memory may not affect taste sensitivity.

A significant decrease in eNos mRNA expression was observed in this study. In previous reports, age-related changes in eNos expression was not found to correlate with activity [22, 23]. Aging-related protein misfolding and aggregation may play critical roles in the pathogenesis of numerous diseases, such as Alzheimer’s disease [24]. Therefore, it is possible that although changes in the mRNA expression levels of the tested genes were not observed, aging affects protein conformation. In future studies, it will be necessary to consider changes in protein conformation due to aging.

In conclusion, we compared whether the effects of aging are observed earlier in taste sensitivity or short-term memory. Changes in taste sensitivity were observed prior to short-term memory decline, suggesting that memory impairment is unlikely to cause a change in taste sensitivity. We believe that our findings will be useful for clarifying the age-related changes in taste sensitivity.

## Supporting information

S1 Table qPCR primer sequences for hippocampus. (DOCX)

S1 Fig Raw data from 7-weeks-old mice. (XLSX)

S2 Fig Raw data from 70-weeks-old mice. (XLSX)

S3 Fig Raw data from 80-weeks-old mice. (XLSX)

S4 Fig qPCR data from the hippocampus of 7 and 80-weeks-old mice. (XLSX)
