# Supplementary material for: Comparison between the timing of the occurrence of taste sensitivity changes and short-term memory decline due to aging in SAMP1 mice
Source: PLoS One. 2021 Mar 23;16(3):e0248673. doi: 10.1371/journal.pone.0248673 (PMC7987193; doi:10.1371/journal.pone.0248673)
Supplement: S1 Table — (DOCX) [file pone.0248673.s001.docx]

**S1 Table. qPCR primer sequences for hippocampus.**

| Gene name |  | Sequence (5’–3’) | Accession number |
| --- | --- | --- | --- |
|  |  |  |  |
| *Ampa1* | Forward | AAAAAGGAGAGGCTGGTGGT | NM_001113325 |
|  | Reverse | CGATGCCGTTCTTTTCTAGC |  |
|  |  |  |  |
| *Ampa2* | Forward | TCAAAATAGCCCAGTGGGAG | NM_001083806 |
|  | Reverse | AACAGGGTTTGATGTGGAGC |  |
|  |  |  |  |
| *Ampa3* | Forward | GAAATGAGCGCTATGAAGGC | NM_016886 |
|  | Reverse | CCATATTTCCCATCACCGAC |  |
|  |  |  |  |
| *Ampa4* | Forward | TACATTGGTGTCAGCGTGGT | NM_019691 |
|  | Reverse | CACTGGGTCCTTCTTTTCCA |  |
|  |  |  |  |
| *Activin βA* | Forward | GAGGAAATGGGCTTAAAGGG | X69619 |
|  | Reverse | TGCTGGACACTGGAAAGATG |  |
|  |  |  |  |
| *Bdnf* | Forward | GTGGTGTAAGCCGCAAAGA | NM_001048139 |
|  | Reverse | AACCATAGTAAGGAAAAGGATGGTC |  |
|  |  |  |  |
| *Creb* | Forward | GGCCTGCAGACATTAACCAT | NM_133828 |
|  | Reverse | TCCATCAGTGGTCTGTGCAT |  |
|  |  |  |  |
| *eNos* | Forward | GGCTCCCTCCTTCCGGCTG | NM_008713 |
|  | Reverse | TCCCGCAGCACGCCGAT |  |
|  |  |  |  |
| *Gdnf* | Forward | TCCAACTGGGGGTCTACG | NM_001301332 |
|  | Reverse | GACATCCCATAACTTCATCTTAGAGTC |  |
|  |  |  |  |
| *Gfra1* | Forward | GCGTGTGAAGCACTGAAGTC | NM_010279.3 |
|  | Reverse | GGTTCAGTTCCGACCCAAC |  |
|  |  |  |  |
| *Gp91phox* | Forward | TGGGATCACAGGAATTGTCA | NM_007807 |
|  | Reverse | CTTCCAAACTCTCCGCAGTC |  |
|  |  |  |  |
| *IL-6* | Forward | CCACTTCACAAGTCGGAGGCTTA | NM_031168.2 |
|  | Reverse | GCAAGTGCATCATCGTTGTTCATAC |  |
|  |  |  |  |
| *iNos* | Forward | GTCACCTACCGCACCCGAG | NM_010927 |
|  | Reverse | GCCACTGACACTTCGCACAA |  |
|  |  |  |  |
| *Ngf* | Forward | TCTATACTGGCCGCAGTGAG | NM_001112698 |
|  | Reverse | GGACATTGCTATCTGTGTACGG |  |
|  |  |  |  |
| *Ngfr* | Forward | ACTGAGCGCCAGTTACGC | NM_033217 |
|  | Reverse | CGTAGACCTTGTGATCCATCG |  |
|  |  |  |  |
| *Mcp-1* | Forward | TTAACGCCCCACTCACCTGCTG | NM_011333 |
|  | Reverse | GCTTCTTTGGGACACCTGCTGC |  |
|  |  |  |  |
| *p22phox* | Forward | TGGCTACTGCTGGACGTTTCAC | NM_007806 |
|  | Reverse | CTCCAGGAGACAGATGAGCACAC |  |
|  |  |  |  |
| *p47phox* | Forward | GTCCCTGCATCCTATCTGGA | NM_001286037 |
|  | Reverse | GGGACATCTCGTCCTCTTCA |  |
|  |  |  |  |
| *p67phox* | Forward | CAGACCCAAAACCCCAGAAA | NM_010877 |
|  | Reverse | AGGGTGAATCCGAAGCTCAA |  |
|  |  |  |  |
| *Tnf-α* | Forward | CGAGTGACAAGCCTGTAGCC | NM_013693 |
|  | Reverse | GGTGAGGAGCACGATGTCG |  |
|  |  |  |  |
| *Ntrk2* | Forward | TGCCCAGAGCAGGATAAGAT | NM_001025074 |
|  | Reverse | AAAGTCCTTGCGTGCATTGT |  |
|  |  |  |  |
| *Vesl-1S* | Forward | TTCACAGGAATCAGCAGGAG | AB019478 |
|  | Reverse | TGTGTCACATCGGGTGTTCT |  |
|  |  |  |  |
